# Poststroke eHealth Technologies–Based Rehabilitation for Upper Limb Recovery: Systematic Review

**DOI:** 10.2196/57957

**Published:** 2025-03-04

**Authors:** Margherita Rampioni, Sara Leonzi, Luca Antognoli, Anna Mura, Vera Stara

**Affiliations:** 1 Innovative Models for Aging Care and Technology IRCCS-INRCA, Istituto Nazionale di Riposo e Cura per Anziani Ancona Italy; 2 Neuroscience Institute University Miguel Hernández Elche Spain

**Keywords:** stroke, rehabilitation, technology-based interventions, upper limb, technologies-based rehabilitation, limb, systematic review, cerebral vascular diseases, patient, effectiveness, database, therapy, conventional therapy, mobile phone

## Abstract

**Background:**

Stroke is one of the most common cerebral vascular diseases, usually affecting people aged 60 years and older. It leads to a variety of disabilities requiring motor and cognitive rehabilitation. Poststroke rehabilitation is critical for recovery, particularly for upper limb impairments, which affect approximately 80% of stroke survivors. Conventional rehabilitation often faces barriers such as cost, accessibility, and patient adherence. In contrast, eHealth technologies offer a promising alternative by providing accessible, cost-effective, and engaging rehabilitation solutions.

**Objective:**

While numerous systematic reviews have explored various aspects of technology-based rehabilitation for poststroke upper limb recovery, there is a notable lack of comprehensive synthesis of these findings. This gap presents challenges, primarily due to the focus on specific technologies, which complicates understanding the overall effectiveness of these interventions. Consequently, clinicians and researchers may find it difficult to assess the field holistically, potentially hindering informed decision-making in clinical practice. This review synthesizes evidence from systematic reviews evaluating the effectiveness of eHealth technology–based interventions for upper limb recovery in poststroke individuals. Two main questions are examined: (1) Are eHealth technology–based therapies more or equally effective than conventional therapies for stroke rehabilitation? (2) What are the main clinical considerations for low-cost eHealth technology–based rehabilitation?

**Methods:**

Comprehensive literature searches were conducted in PubMed, Web of Science, Scopus, Embase, and Google Scholar using predefined inclusion criteria based on the Population, Intervention, Comparison, Outcome, and Study Design (PICOS) framework. Systematic reviews published in English without date restrictions were included. The PRISMA (Preferred Reporting Items for Systematic Reviews and Meta-Analysis) flowchart guided study selection. Methodological quality was assessed using the Assessment of Multiple Systematic Reviews (AMSTAR 2) criteria.

**Results:**

A total of 1792 records were screened, resulting in 7 systematic reviews published between 2019 and 2023 being included. These reviews encompassed 95 studies involving 2995 participants with a mean age of 58.8 years across acute, subacute, and chronic stroke phases. Interventions included telerehabilitation, mobile health (mHealth) apps, augmented reality (AR), virtual reality (VR), wearable devices, and exergames. While AR and VR demonstrated potential benefits when combined with conventional therapies (eg, AR showing significant improvements in upper limb function with a standardized mean difference 0.657; *P*<.001), evidence for stand-alone effectiveness remained inconclusive due to heterogeneity in study designs, intervention protocols, and outcome measures. Most reviews were rated as critically low quality due to methodological limitations.

**Conclusions:**

eHealth technologies hold promise for enhancing upper limb rehabilitation post stroke by addressing barriers such as cost and accessibility while providing engaging interventions. However, the field remains fragmented with insufficient evidence to establish clear efficacy. Future research should focus on standardizing protocols, optimizing neurorehabilitation principles such as dosage and task specificity, and improving methodological rigor to evaluate these interventions’ long-term impact better.

## Introduction

### Background

A stroke event refers to the alteration in brain functions after a sudden disruption of brain blood flow. It has an incidence of 15 million people a year. Stroke is one of the most common cerebral vascular diseases, usually affecting the population aged 60 years and older, leading to a variety of disabilities for about 5 million surviving patients who require motor and cognitive rehabilitation [1,2].

This massive incidence puts tremendous pressure on health care systems to satisfy the need for effective and sustainable solutions for rehabilitation poststroke after hospital discharge. Indeed, the consequences of stroke encompass a spectrum of disabilities, spanning physical impairments to cognitive challenges, including issues with language, social interactions, and emotional well-being [3]. In about 80% of cases, patients with stroke experience motor impairments of the upper limbs [4], which are functionally complex and difficult to recover, making activities of daily living very difficult for the patients. Poststroke disability negatively impacts the quality of life and health, and only 25% of patients recover with only minor impairments [5].

Poststroke rehabilitation has a lead role in the recovery of the patients, and it is never too late to start [6]. The level of recovery varies from person to person, but with time, effort, and support, many patients with stroke can significantly improve their function and quality of life [7]. For this, stroke rehabilitation should be planned and implemented in a structured and coordinated approach to help patients regain their physical, cognitive, and functional abilities. For instance, the increasing understanding of brain plasticity is relevant to the rehabilitation strategy and outcome after brain damage, and the principles of experience-dependent plasticity are valuable for treatment [8,9]. Indeed, rehabilitation requires multiple and varied therapeutic approaches at any stage of disease (acute and chronic phases) [10]. In addition, the quantity and quality of treatment are important for an effective recovery: rehabilitation after a stroke should be task-oriented, offered in large doses, and with an active learning component for providing intentional and more effective training [11]. Training several times daily is necessary to exploit the neuronal plasticity that ensures effective neurorehabilitation.

Nevertheless, high-intensity treatments are very expensive for the health care system, and hospital stays often must be reduced. This could mean that patients could still have functional deficits on the day of hospital discharge [4], especially in the upper limb [11,12].

In addition, outpatient therapy is not feasible for many patients because of the high costs of individual specialized therapies and the logistical difficulties of transportation from or to the hospital.

Home rehabilitation is a good option to continue recovery from chronic diseases such as stroke and a good alternative to manage long-term rehabilitation problems. Therefore, home rehabilitation is necessary to provide such a large amount of training [13] because traditional rehabilitation programs do not usually comply with all the abovementioned requisites. Thus, to maintain progress in the recovery process, the conventional rehabilitation pathway must include self-administered home exercises, a home rehabilitation solution, and outpatient therapy.

However, even within these strategies, certain limitations exist. Patients often lack motivation or lose interest in performing exercises independently, for example, they may consider them too difficult or too easy or monotonous. Here, technology can play a crucial role. The absence of external feedback can be overcome with interactive systems that provide real-time guidance and encouragement, stimulating patients to continue their rehabilitation [14].

Technology plays a crucial role in bridging the gap between home rehabilitation and remote therapy. By providing tools and platforms, it enables patients with stroke to receive therapy, guidance, and support without the need for a physical presence in a health care facility. This not only improves long-term recovery but also allows hospitals to reduce the length of outpatient treatment and its associated costs, while maximizing the treatment capacity of therapists.

### Technology That Impacts Poststroke Rehabilitation

Attempting to reduce and overcome all the mentioned logistical and economic barriers of long-term rehabilitation, technological solutions offer a beneficial and effective alternative to conventional therapy, making rehabilitation more accessible to everyone. Rehabilitation technologies can significantly enhance the effectiveness and accessibility of stroke rehabilitation programs when incorporating principles of experience-dependent plasticity [8,9,15]. These advanced and simple technologies are more commonly explored and used in developed countries, mainly due to their readiness and availability, but their potential for accessibility is universal, making them a promising solution for poststroke rehabilitation worldwide [16].

Advanced technological interventions for poststroke rehabilitation include robotics, transcranial magnetic stimulation, transcranial direct current stimulation, brain-computer interface, and functional electrical stimulation. These therapies, while effective, require a certain level of expertise and resources that may not be readily available in a home setting. Therefore, they are typically conducted in a clinical or rehabilitation center under the supervision of trained professionals. Other current less invasive technologies are virtual reality (VR), augmented reality (AR), and activity trackers (such as accelerometers, gyroscopes, pedometers, breath sensing, heart rate monitors, and calorie trackers) [17]. As a cutting-edge and computer-generated simulation technology, VR can create an enriched environment, facilitate task-specific training, and provide multimodal feedback to augment functional recovery [18]. Users can use their hands or movement sensors, such as gloves or joystick, to interact with virtual objects. The development of low-cost sensors has allowed this technology to spread at the consumer level: the Sony PlayStation 3, Microsoft Xbox 360, and Nintendo Wii are examples of VR-based consoles [15]. AR technology combines real and virtual objects to provide an interactive real-time experience in a common environment [19]. Users can interact with AR through devices such as smartphones, visors, display, or active mirrors. These devices use cameras, sensors, and software to overlay digital information onto the real world. In contrast, functional electrical stimulation or neuromuscular electrical stimulators have been used predominantly for stimulating lower and upper extremity functions [20].

Telerehabilitation (TR), mobile health (mHealth) apps, assistive technologies, and electromechanical gait training (with variable automated speed and sensing treadmill) are simple technological interventions for poststroke rehabilitation [17]. Home-based TR is a branch of telemedicine that consists of the use of a variety of telecommunication platforms (such as telephone visits, mobile apps, serious games, web-based self-care programs, web-based videoconferencing, and sensor-based telemonitoring) by health care professionals to provide necessary patient care and remote evaluation, supervision, and support for persons with disabilities living at home [11,21]. Home-based TR allows to meet the rehabilitation needs of stroke survivors living in rural areas with limited health services, especially in westernized countries, where the stroke burden is rapidly increasing [22]. Home-based TR implies access at any time and from any place to rehabilitation services to address stroke aspects: (1) *motor function*: upper and lower extremities, balance, and gait; (2) *cognitive function*: spatial neglect, cognition, and memory; and (3) *language*: aphasia [23]. TR can be delivered synchronously or asynchronously, depending on patients’ needs, medical conditions, and treatment plans [24]. The choice between synchronous and asynchronous TR depends on various factors, including the patient’s needs, the nature of the therapy, and the available technology. Synchronous sessions are often used for live consultations, real-time feedback, and interactive exercises. Asynchronous sessions are more flexible and beneficial when patients need rehabilitation in their daily routines. Some TR programs use a combination of both approaches to offer a well-rounded service that combines real-time interaction with flexibility and convenience.

Also, mHealth apps are defined as health and well-being mobile services for medical care delivered using a mobile app or other wireless technology. These are interesting for their mobility, multifunctional skills such as reminders and videos, and ability to support specific rehabilitation goals and promote self-management [25]. mHealth apps for stroke rehabilitation can target different aspects of the disease, and there are mobile apps designed as games to improve finger dexterity and programs to increase adherence to home rehabilitation exercises, for example, for upper limb rehabilitation [26,27].

### Objectives

This study identifies and appraises published systematic reviews. The aim is to describe the quality, summarize and compare the conclusions, and discuss the strengths and weaknesses in the effectiveness of low-cost eHealth technology–based rehabilitation for the recovery of the upper limb in poststroke individuals.

While numerous systematic reviews have examined various aspects of technology-based rehabilitation for poststroke upper limb recovery, there is a lack of comprehensive synthesis of these findings. This gap presents several challenges. The first challenge concerns the focus on specific technologies, which makes it difficult for clinicians and researchers to understand the overall effectiveness of technology-based interventions and the field holistically. Without this comprehensive understanding, health care providers may struggle to make informed decisions about implementing these interventions in clinical practice. We aim to address these gaps by reviewing systematic reviews and providing a comprehensive, high-level synthesis of the current evidence.

This systematic review focuses on technology-based rehabilitation with interventions supported by synchronous (real-time care) or asynchronous (not real-time care) TR, mHealth apps, or eHealth portable devices equipped with VR or AR apps. Hence, in this systematic review, we assessed the low-cost eHealth systems to determine their impact on improving upper limb functional recovery among patients with stroke by examining 2 main questions: (1) Are eHealth technology-based therapies more or equally effective than conventional therapies for stroke rehabilitation? (2) What are the main clinical considerations for low-cost eHealth technology–based rehabilitation?

## Methods

### Databases, Criteria, and Search Strategy

Separate literature searches were conducted in PubMed, Web of Science, Scopus and Embase databases, and Google Scholar. The Population, Intervention, Comparison, Outcome, and Study Design (PICOS) framework was used to define the following inclusion criteria: (P) the target population was composed of persons with poststroke, (I) we considered technological interventions for upper limb rehabilitation in stroke survivors (ie, eHealth rehabilitation, at home TR, and smartphone-based rehabilitation), (C) studies were selected with or with no control group comparison, (O) we considered upper limb function recovery outcomes, and (S) we search for systematic reviews written in English that evaluated the effectiveness of technology-based intervention for the rehabilitation of the upper limb in poststroke patients. There was no restriction on publication dates. The combination of key terms reported in Multimedia Appendix 1 was used for the search in each database. The searches were finalized in May 2023. Papers were excluded if they were not systematic review papers or were not written in English.

According to the predefined criteria, the screening phase was based on analyzing titles and then abstracts. Later, full-paper articles of those titles or abstracts of screened publications were reviewed independently by MR and SL. VS was involved in reaching a consensus in cases of disagreement. Studies that met the inclusion criteria were included, and the results of the searches were summarized. We used the PRISMA (Preferred Reporting Items for Systematic Reviews and Meta-Analysis) [28] flowchart in the retrieval and selection process. Each included systematic review was summarized by extracting essential data to answer the 2 research questions. The themes analyzed are (1) types of interventions and technology used, (2) effectiveness of eHealth technology–based interventions for recovering the upper limb, and (3) methodological quality assessments using the Assessment of Multiple Systematic Reviews (AMSTAR 2) criteria. The summaries were synthesized to provide a cohesive narrative highlighting similarities and differences across studies. This process involved comparing intervention types (eg, VR, AR, and mHealth apps), their reported effectiveness, and any noted limitations or gaps in evidence.

### Study Quality Assessment

Three authors independently and anonymously appraised the final papers’ methodological quality using the AMSTAR 2 [29]. AMSTAR 2 is designed to appraise systematic reviews of health care interventions and rate their overall confidence. The tool can appraise various aspects of systematic reviews of randomized controlled trials (RCTs), nonrandomized studies of interventions, or both.

It is composed of 16 items evaluated either with “yes” or “no” (items 1, 3, 5, 6, 10, 13, 14, and 16); with “yes,” “partial yes,” or “no” (items 2, 4, 7, 8, and 9); or with “yes,” “no,” or “no meta-analysis conducted” (items 11, 12, and 15). This tool evaluates the overall quality based on performance in critical and noncritical domains, which are assigned different weights in the rating rules. A “yes” answer means the item is fulfilled and is considered a positive result. Based on the performance in these 16 domains with different weights, overall ratings were generated, and the quality was determined to be “high,” “moderate,” “low,” or “critically low.” Namely, high confidence was defined as no or 1 noncritical weakness. Moderate confidence was defined as more than 1 noncritical weakness. Low confidence was defined as 1 critical flaw with or with no noncritical weaknesses. Critically low confidence was defined as more than 1 critical flaw with or with no noncritical weaknesses. Each review was appraised by 3 authors (MR, SL, and VR). In case of disagreement, it was planned that a fourth author (LA) would have solved them, but this was not the case. The analysis is reported in Multimedia Appendix 2.

## Results

### Characteristics of the Selected Reviews

**Figure 1:**
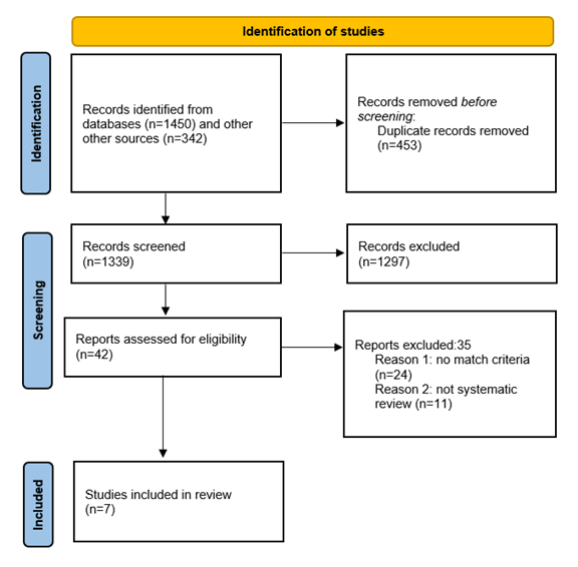
The PRISMA (Preferred Reporting Items for Systematic Reviews and Meta-Analysis) flowchart.

As reported in the PRISMA flowchart (Figure 1), 7 systematic reviews were included. A summary of the studies and their findings is reported in Multimedia Appendix 3. Initially**,** the search process identified 1450 records from the databases and an additional 342 by Google Scholar. After duplicates (n=453) were removed, 1339 papers remained for initial screening by title. From this process, other 1297 papers were excluded because (1) studies involved patients for general rehabilitation (n=18), (2) the intervention was not specific for upper limb recovery functions (n=195), (3) the intervention was performed using other technologies (n=402), and (4) studies dealt with other topics or disorders (n=682). This process resulted in 42 potentially eligible abstracts. The authors analyzed the retained abstracts to obtain the final list of full-text papers to be reviewed. After examining the abstracts, 24 were excluded, as they did not fit the established criteria of the target population and the specific technology-based intervention. A second screening step was performed for those full-text papers that matched all the criteria (n=18). The other 11 were excluded from this process because the type of studies were not systematic reviews. The 7 studies included were published between 2019 and 2023.

Five of the selected reviews defined the study’s design using the PICOS framework [30-34] whereas the other 2 defined the inclusion criteria [35,36]. Four reviews [30,31,35,36] were registered in the research protocol on the international database of prospectively registered systematic reviews in health and social care (PROSPERO). A total of 95 studies were included in the 7 reviews that overall mapped not only RCTs [30,32,33,35,36] and RCTs and non-RCT studies [31,34] but also observational studies [32] and uncontrolled clinical trials [34]. A total of 2995 patients were enrolled with a mean age of 58.78 years (SD 0.15 years). The average duration of stroke onset among the participants ranged from 7 days to more than 5 years, as each of the 7 reviews included patients in the acute (7 days to 3 months), subacute (3-6 months), and chronic (>6 months) phases. Only 1 paper also included patients without specifying stroke duration [35].

The chronic patients enrolled have had a stroke from 6 months or more [33,36], 21 months [35], 3 years [30,34] to 5 years [31], and more than 5 years [32].

Four of the selected reviews [30-32,34] investigated the effectiveness of technology-based rehabilitation interventions on physical functioning compared with a combination of traditional treatments in patients with stroke [30], upper limb wearable technology for improving physical activity and social participation in adult stroke survivors [31], AR for the upper and lower limb functional recovery after stroke [32], and mHealth apps containing a physical training in stroke rehabilitation [34]. Two reviews [33,35] examined the effects of home-based exergaming interventions on upper limb activity after stroke, compared with conventional therapy, in postintervention and follow-up [33]; the use of mobile apps for stroke rehabilitation on stroke-related impairments (motor paresis, aphasia, and neglect); and functional outcomes (adherence to exercise, activities of daily living, quality of life, secondary stroke prevention, and depression and anxiety) [35]. Only 1 review [36] gathered evidence on VR-based TR for patients after stroke and compared it with conventional in-person rehabilitation.

As reported in Table 1, all 7 reviews analyzed the upper limb function as an outcome. The categorization of the most used outcome measures of upper limb is reported in Multimedia Appendix 4.

**Table 1:** Outcomes.

Outcomes (upper limb function)	Authors (years)						
	Rintala et al (2019) [30]	Parker et al (2020) [31]	Phan et al (2022) [32]	Gelineau et al (2022) [33]	Rintala et al (2023) [34]	Szeto et al (2023) [35]	Hao et al (2023) [36]
Spasticity	N/A^a^	√	N/A	N/A	N/A	N/A	√
Pain	√	√	√	N/A	√	N/A	√
Strength	√	√	√	N/A	√	√	√
Difficulty perceived	√	√	√	√	N/A	N/A	√
Upper extremity function	√	√	√	√	√	√	√
Motor impairment	√	√	√	√	√	√	√
Fine manual dexterity	√	√	√	√	√	√	√
Gross manual dexterity	√	√	√	√	√	√	√

^a^N/A: not applicable.

Five reviews also assessed lower limb function and walking [30,32,34-36], balance [30,32,34,36] and physical activity, and function [30,34]. Participation was included as an outcome in 3 of the selected reviews [30-32], while the other 3 [34-36] evaluated the quality of life. Three studies assessed the activity of upper limb function [31-33], lower limb function [32], and participation [31,32] after stroke, according to the World Health Organization’s International Classification of Functioning, Disability and Health framework [37].

### Types of Interventions and Technology Used

Different low-cost eHealth technology–based interventions were reported in the 7 reviews analyzed. Web-based video monitoring and physical home exercises use smartphones, tablets, and web-based programs for real-time therapist-patient interaction and feedback. mHealth apps aim to improve limb movement and dexterity through user interaction with the app, allowing therapists to preselect exercises and assist caregivers in monitoring patient progress. Wearable technologies such as sensors and accelerometers capture actions or measurements and relay information for analysis and are connected to video games that require active body movements. AR projects virtual objects in real environments, allowing patients to interact with them and receive feedback. VR creates immersive simulations that require patient interaction, often with remote or in-person therapist supervision.

#### Web-Based Video

Web-based video monitoring and techniques for monitoring physical home exercises, goal settings or overall treatment, gamification, and accelerometers are mapped in 4 of the selected reviews [30,33-35]. The tools used varied from smartphones, phones, tablets, DVDs and programs via the web [30], and video game environments that required active body movements to control the game [33], to mHealth apps [34,35].

Looking at the details of the selected reviews, web-based video monitoring, phone calls, and messaging are the most common technologies used till now, as mentioned by Rintala et al [30]. They normally ensure a real-time therapist-patient interaction, with a “call” frequency ranging from 3 to 5 times per week to 1 per month. The therapist can also provide feedback through the web and, when necessary, by scheduling virtual training (eg, exercise videos) in advance [30].

Another option for remote technology–based rehabilitation, analyzed by Gelineau et al [33], is to provide exergames requiring a physical interaction (active body movements) to play the game. In that case, nonspecific video game systems (eg, Nintendo Wii, Xbox Kinect, etc) need to be combined with specific rehabilitation systems (eg, Rehabilitation Gaming System, virtual gloves, etc) or specific rehabilitation devices (eg, Hand Mentor Pro, Polhemus 3, etc). Patients with a prescription, usually ranging from 3 to 7 days per week, can use exergames systems independently, producing self-reported or observational measures (eg, technological sensors and therapist telehealth visits) [33].

#### mHealth Apps

Leveraging everyday technologies, such as smartphones and tablets or PCs, has become increasingly prominent in rehabilitation. This is made possible through implementing mHealth apps well investigated in the study by Gelineau et al [33]. These apps incorporate physical training components and offer a customizable approach tailored to individual patient requirements. This customization encompasses specific goals, desired difficulty levels, and the duration of app usage.

Moreover, these apps serve various purposes, including interactive gaming (eg, FINDEX and ARMStrokes), prescription of exercise routines (eg, CARE4STROKE), and progress monitoring (eg, STARFISH). They can also be integrated with devices such as inertial measurement unit sensors and pedometers to enhance their effectiveness. This innovative use of technology complements traditional physiotherapy and widens the spectrum of rehabilitation possibilities.

Generally, the “gaming apps” require limb movement and interaction, aiming, for example, to improve upper limb and finger dexterity. In the “exercise prescription” app a therapist preselects a set of standardized exercises shown in the app, allowing the caregiver assistance, if necessary. “Monitoring apps” supervise and check patients’ physical behavior (eg, number of steps per day, walking or sitting time, walking distance or speed, etc).

For all the types of apps, the prescription is maximally 30 minutes per session, from 1 to 7 times per week. The therapist’s constant presence is not necessary, thanks to auditory and vibration feedback provided directly by the apps [34].

The mobile apps can be on any operating system (iOS, Android, and Windows) and on any aspect of stroke impairment rehabilitation (motor paresis, aphasia, and neglect). Focusing more on their possible goals, we could have therapy apps (with users’ active device interaction to complete activities) or rehabilitation videos (exercises mobile guide), education apps (to learn about stroke), reminders (messaging to encourage compliance), or even a combination of them [35].

#### Wearable Technologies

Parker et al [31] described 2 types of wearable devices worn by patients. The first device operated independently and functioned as a central connector for other devices, whereas the second one captured specific action or executed a measurement and then sent the information to a primary wearable device for analysis.

The “wearable technologies” are something portable to wear externally on the body and to be used independently of a therapist. They can be used in both clinical and nonclinical settings to facilitate recovery, provide formative and real-time feedback, and measure intervention outcomes over long periods of time. They span from microelectromechanical systems (with accelerometers, gyroscopes, etc) to electromyographic biofeedback to robotics. This type of intervention can last 3-12 weeks, with various intensities [31].

#### AR- and VR-Based Rehabilitation

AR- and VR-based rehabilitation systems providing games were reviewed by Phan et al [32] and Hao et al [36]. Such systems included using head-mounted displays, Leap Motion, HD webcam, Microsoft Kinect v2 sensors connected to a laptop, or AR mirrors with visual tracking methods. AR technology is a real-time projection of virtual objects or scenarios in a common and real environment or place. During the game, the patients will interact with these virtual objects and receive automatic instructions, score, and visual and audio feedback via tracking devices (mouse, arm skate, HD Webcam, etc). The AR systems usually allow the creation of a personal training program with various training intensities, and the treatment duration is between 30 minutes and 1 hour per session [32]. VR is a computer-generated simulation technology that requires patient interaction, providing multimodal feedback. VR systems are often installed at patients’ houses and may include the remote supervision of a therapist (eg, virtual-based TR group, interfaces for patient, and therapist remote communications). It is also used at hospitals or clinics, with a therapist’s in-person supervision or instruction [36].

### Effectiveness of eHealth Technology–Based Interventions for Recovering the Upper Limb

The selected reviews were analyzed regarding the effectiveness of the low-cost eHealth interventions. An effective intervention is based on statistically significant differences in 1 or more outcomes from the baseline, compared with the control, or if there is no control comparator, compared with another SD. Although all technology-based interventions included in these studies are economically favorable rehabilitation delivery models, no clear or direct evidence of impact on function recovery has been underlined.

Parker et al [31] highlighted little evidence to support the use of wearable technologies to improve activity and participation in the recovery of the upper limb.

Even if all the other reviews [30,32-36] reported similar effectiveness to conventional treatments, results are interpreted and discussed cautiously. For example, Rintala et al [30,34], Gelineau et al [33], and Szeto et al [35] discussed that rehabilitation technology, including mHealth apps, may have benefits as an additional treatment, but a lack of robust evidence does not consent authors to determine apparent effects.

Also, in the case of AR-based [32] and VR-based [36] TR, patients might still have a similar subjective experience of rehabilitation with therapists’ supervision as in-person rehabilitation. According to Phan et al [32], the use of AR significantly influenced the upper limb function (standardized mean difference 0.657, 95% CI 0.287-1.026; *P*<.001). AR-based applications could offer options for increasing treatment intensity and promoting motor recovery after a stroke used with conventional rehabilitation methods. Also, for Hao et al [36], VR-based TR is a promising avenue for patients with stroke that can potentially overcome the barriers of traditional in-person rehabilitation. VR-based TR achieved comparable outcomes in the upper extremity function and equivalent effects on balance ability compared with in-person rehabilitation.

One aspect that holds significant clinical relevance is the duration of the intervention, which is measured in terms of both the quantity and the intensity of the dosage. Studies suggested a positive correlation between the time allocated for therapy and the therapy outcomes [38]. In the 7 reviews, the duration of the trials primarily ranged from 2 to 12 weeks.

According to Parker et al [31], improvements were observed across some studies for the control and intervention groups. The increase in the amount of rehabilitation has led to improvements in all the studies analyzed. This observation could suggest that a fundamental mechanism for improvement is the augmentation of the amount of rehabilitation administered. This concept has been acknowledged and incorporated into the national clinical guidelines for stroke. Szeto et al [35] concluded that the dosage and duration should be customized to address individual problems.

Some studies included in the study by Gelineau et al [33] reported follow-ups. However, in most of these cases, the duration of the follow-up is too short (4-24 weeks), leading to inconsistent results.

The selected reviews did not mention or report rigorous application of the experience-dependent plasticity principles, which are not always made explicit or clear [8,9].

In conclusion, further research is necessary to differentiate between the mechanisms of dosage and intensity. This will aid in understanding the impact of the volume of rehabilitation activity and how it compares with the intensity, which is the amount of rehabilitation administered over a specific time of period.

### Quality Assessment

Among the 7 reviews assessed, 6 were rated as critically low quality [31-36], and 1 as low quality according to AMSTAR-2 criteria (Multimedia Appendix 2). Key issues affecting the validity of these reviews included inadequate protocol registration, inconsistent risk of bias assessments due to poor reporting on blinding and allocation concealment, and substantial heterogeneity across studies.

Only 1 review [36] lacked an explicit statement about protocol registration before the study began. All reviews demonstrated partial adequacy in their literature searches, with written protocols or guides that included review questions, search strategies, inclusion or exclusion criteria, and risk of bias assessments. None justified the exclusion of individual studies.

For risk of bias assessment, only 1 review [36] failed to use a satisfactory technique, while another [35] partially excluded nonrandomized studies of interventions. Three reviews [31,34,35] did not conduct a meta-analysis and 1 omitted it [32]. Five reviews [30,31,33-35] considered the risk of bias in their discussions. Only 2 reviews [30,32] adequately investigated publication bias and its potential impact.

In noncritical domains, 2 reviews [35,36] did not include all PICO components. All reviews explained their study design selections and performed study selection in duplicate; however, only 3 [30,31,33] duplicated data extraction. All reviews adequately described included studies; 4 [30,33,34,36] met all required criteria. Only 1 review reported funding sources for included studies. While 3 reviews [31,34,35] did not conduct a meta-analysis, only 2 [30,33] assessed the impact of bias on meta-analysis results. Five reviews [30,32-34,36] satisfactorily explained observed heterogeneity. Only 1 review failed to report potential conflicts of interest [30].

Overall, the critically low and low-quality assessments of the 7 reviews reflect the methodological shortcomings reported across these studies as low to moderate due to methodological limitations such as small sample sizes, lack of blinding, and heterogeneity in interventions.

## Discussion

### Principal Findings

Low-cost eHealth technology–based interventions should provide affordable and scalable science-based TR to cope with the pressure on health care systems to satisfy the need for effective and sustainable solutions for rehabilitation post stroke after hospital discharge.

In this study, 7 systematic reviews were selected to map the actual scenario of the use of low-cost eHealth technology–based interventions by examining two main questions: (1) Are eHealth technology–based therapies more or equally effective than conventional therapies for stroke rehabilitation? (2) What are the main clinical considerations for low-cost eHealth technology–based rehabilitation?

Most of these studies used advanced technologies with AR- and VR-based technologies and simple technologies with TR, mHealth apps, assistive technologies, and gait training.

Despite the differences among the reviews, all reached a unanimous conclusion, highlighting a cautious interpretation of data due to unclear evidence of the effectiveness of the examined technologies in restoring lost upper limb function [30-36]. Indeed, the variability in interventions, study designs, participant demographics, and measured outcomes contributed to heterogeneity across the studies, underscoring the need for future research to establish rehabilitation intervention principles that can inform the development of targeted innovative technologies [30-36]. Studies indicated that AR, VR, wearable devices, and exergames can improve upper limb motor function when combined with conventional rehabilitation methods, but their effectiveness alone is often comparable with traditional therapy [30,32-34,36].

Different clinical considerations favoring low-cost eHealth technology–based rehabilitation can be derived from the analyzed reviews. A central consideration is the potential of technology-driven interventions to enhance at-home poststroke rehabilitation for motivating survivors through interactive, user-friendly, engaging, and cost-effective tools. Indeed, eHealth systems could be seen as a way to reduce and overcome logistical and economic barriers of long-term conventional rehabilitation approaches by providing engaging and motivating rehabilitation interventions at home. Even if cognitive impairments or a lack of familiarity with technology can hinder the adoption of eHealth solutions, systems must be user-friendly to accommodate stroke survivors with varying levels of ability [30]. Continued research into the usability and effectiveness of this kind of system can guide the designers in developing a more user-friendly and engaging virtual ambient. Moreover, health care institutions should invest in patient education and engagement by introducing innovative systems into clinical practice.

Recent literature suggests that stroke affects the entire brain and its network properties, making it a network disease. Therefore, stroke-related neurological deficits and their recovery depend on neural network interaction patterns and follow principles of network plasticity [39]. In addition, the observation that network interactions are correlated with current and future neurological function directly leads to whether their modulation through therapy might be feasible and clinically useful [40]. This evidence could open new frontiers for developing technology-based rehabilitation in poststroke individuals.

The feasibility of modulating brain networks through technology-based therapy may depend on several factors inherently linked to neurorehabilitation principles. According to these principles, exposure to specific training experiences in a recovery pathway improves impairment precisely because of the activation of these mechanisms of neuronal plasticity and remodeling [8]. The following neurorehabilitation principles should guide the selection of training experiences and rehabilitation technologies to optimize effectiveness [15]: massed practice, spaced practice, dosage, task-specific practice, goal-oriented practice, variable practice, increasing difficulty, multisensory stimulation, rhythmic cueing, explicit feedback or knowledge of results, implicit feedback or knowledge of performance, modulate effector selection, action observation or embodied practice, motor imagery, and social interaction. On the contrary, the selected reviews did not consistently adhere to all the principles of experience-dependent plasticity [8]. For example, interventions involving web-based video monitoring and phone calls often lack clear dosage specifications [30]. The AR system analyzed by Phan et al [32] used specific sensors [41] and viewers [42] to deliver exercises through games in both clinical and home settings. This system incorporated the neurorehabilitation principles of feedback (eg, game scores and social interaction), variable practice, and training intensity. However, AR systems face limitations: they are not always user-friendly, portable, or low-cost. While they offer a viable rehabilitation option, they may not be the most convenient solution for home-based upper limb rehabilitation.

Gelineau et al [33] highlighted the heterogeneity in exergame-based rehabilitation regarding the supervision and treatment dosage. Exergames are promising technologies that support stroke recovery and TR but still need to be explored more from clinical and neurorehabilitative perspectives. mHealth apps studies [34,35] often did not specify dosage prescriptions, indicating a limited application of neuroscience principles.

In VR-based TR [31,36], some systems offer synchronous patient supervision, while others provide only asynchronous settings; both ensure activity feedback and patient supervision. Nevertheless, the dosage prescriptions for these TR systems require further exploration. Low-cost eHealth technologies offer a promising avenue for stroke rehabilitation. They reduce barriers to rehabilitation by enabling remote monitoring and therapy delivery. This is particularly beneficial for patients in rural or underserved areas who face challenges accessing traditional in-person care [30,36]. Technologies such as wearable sensors and off-the-shelf gaming systems (eg, Wii) require poor infrastructure, making them suitable for home use [31,33]. Moreover, wearable devices and sensors allow continuous monitoring of movement quality, intensity, and frequency, providing valuable data for therapists to refine treatment plans remotely [31].

In conclusion, the integration of low-cost eHealth technology–based rehabilitation into clinical practice requires addressing usability challenges, standardizing protocols, rigorous adherence to all the principles of experience-dependent plasticity [8], and ensuring long-term effectiveness through further research. Combining these technologies with conventional therapies appears to yield the most beneficial outcomes for patients with stroke [43,44].

### Limitations

Data sources were drawn only from PubMed, Web of Science, Scopus and Embase databases, and Google Scholar, excluding other data sources. Moreover, it is possible to miss relevant studies, especially if they are published in nonindexed journals, unavailable in electronic databases, or written in languages not included in the search criteria. Even if the PICOS framework was used to define the inclusion criteria, the combination of key terms to target the population and the specific technology-based intervention could have omitted some results from the search. Another significant limitation is the search strategy, which relied on a simple keyword search in titles and abstracts across databases. This approach may have inadvertently excluded relevant studies due to the lack of use of MeSH (Medical Subject Headings) and other controlled vocabulary terms that could have enhanced the comprehensiveness of the search. Medical Subject Headings terms are designed to capture all relevant papers on a topic, regardless of variations in terminology used by different authors. Future reviews should consider incorporating comprehensive search strategies, including keywords and controlled vocabulary terms across multiple fields within each database, to enhance the validity and reliability of conclusions drawn.

The heterogeneity and the different quality levels of the included studies are the significant limitations claimed by almost all the selected reviews [30,31,33-36]. Even if each review assessed quality using Physiotherapy Evidence Database Scale [30,33,34,36], Cochrane Risk of Bias for RCTs [31,35], Downs and Black Instrument for non-RCTs [31,34], and QualSyst [32], given the differences in assessment quality, our reviews’ results should be interpreted and generalized with caution.

Moreover, we restricted the search strategy to only low-cost eHealth technology–based interventions for the upper limb recovery. This decision could have been the reason for excluding other relevant studies [45,46].

### Implications for Clinical Practice and Future Research

This review indicated that the field of technology-based rehabilitation is still fragmented due to poor evidence achieved in terms of efficacy. This is probably due to the high heterogeneity of the experimental studies. We mapped various study designs, outcomes, and quality levels that demonstrate only the potential to assist with stroke recovery and augment face-to-face rehabilitation. Future research is called to frame a more robust methodology with a larger sample size [31-36] and valid measurement tools [31,34], deeper investigation on different gender and age groups [31,32], and other impacts depending on the stroke stage [33,35]. Also, the trial length and the study follow-up time were mentioned [35,36]. Regarding technology readiness level, different issues were underlined to overcome the drawbacks of the reported technology-based rehabilitation interventions, for example, the integration and interoperability with intelligent infrastructure and the design of an attractive, user-friendly, portable, and low-cost system [32,34,35]. So, when developing a technology-based rehabilitation program, it is crucial to carefully plan and link all the relevant actors, user-driven design guidelines, and principles of neuroscience.

With this potential, the preferred option is to better understand the impact of technology interventions on varying types of stroke deficits and related outcomes, both alone and in combination with traditional rehabilitation. As Gelineau et al [33] have already highlighted the need for a gold standard, this field of research could benefit from standardized protocols provided to patients, enabling comparison and interpretation to discover evidence currently missing [15].

### Conclusions

This review pooled the findings of 7 systematic reviews to map the actual scenario of the use of low-cost eHealth technology–based interventions and determine whether (1) eHealth technology–based therapies are more or equally effective than conventional therapies for stroke rehabilitation and (2) what the main clinical considerations for low-cost eHealth technology-based rehabilitation are. It found heterogeneity among interventions and measures, but the commonality of no clear or direct evidence of impact on recovery of function has been underlined. Advanced and simple technologies used for stroke rehabilitation allow overcoming financial, physical, and attitudinal barriers while providing engaging, specific, and low-cost exercises with constant feedback and supervision. These technologies serve as a valuable enhancement to traditional rehabilitation methods, particularly for the upper limb [47]. Unfortunately, demonstrating the efficacy of such interventions for restoring function after a stroke is still a challenge. Combining these technologies with interventions that follow neurorehabilitation principles may be more effective in promoting the recovery and retention of motor and cognitive functions after a stroke. Indeed, the integration of low-cost eHealth technology–based rehabilitation into clinical practice requires addressing usability challenges, standardizing protocols, rigorous adherence to all the principles of experience-dependent plasticity [8], and ensuring long-term effectiveness through further research. Combining these technologies with conventional therapies appears to yield the most beneficial outcomes for patients with stroke [48,49].

## Appendix

Multimedia Appendix 1 Strings used for the search in PubMed, Web of Science, Scopus and Embase databases, and Google Scholar.

Multimedia Appendix 2 Amstar-2 .

Multimedia Appendix 3 Characteristics of the reviewed studies.

Multimedia Appendix 4 Outcome measures.

Multimedia Appendix 5 PRISMA checklist.

## Abbreviations

AMSTAR 2: Assessment of Multiple Systematic Reviews
AR: augmented reality
MeSH: Medical Subject Headings
mHealth: mobile health
PICOS: Population, Intervention, Comparison, Outcome, and Study Design
PRISMA: Preferred Reporting Items for Systematic Reviews and Meta-Analysis
RCT: randomized controlled trial
TR: telerehabilitation
VR: virtual reality

## References

[1] Wu D, Zhang H, Leng Y, Li K, Li S, Lo WLA (2022). A bibliometric analysis of telerehabilitation services for patients with stroke. Front Neurol.

[2] Moradi V, Babaee T, Esfandiari E, Lim SB, Kordi R (2021). Telework and telerehabilitation programs for workers with a stroke during the COVID-19 pandemic: a commentary. Work.

[3] National Institute of Neurological Disorders and Stroke (2020). Post-stroke rehabilitation. NINDS Publications.

[4] Rozevink SG, van der Sluis CK, Garzo A, Keller T, Hijmans JM (2021). HoMEcare aRm rehabiLItatioN (MERLIN): telerehabilitation using an unactuated device based on serious games improves the upper limb function in chronic stroke. J Neuroeng Rehabil.

[5] Virani SS, Alonso A, Benjamin EJ, Bittencourt MS, Callaway CW, Carson AP, Chamberlain AM, Chang AR, Cheng S, Delling FN, Djousse L, Elkind MS, Ferguson JF, Fornage M, Khan SS, Kissela BM, Knutson KL, Kwan TW, Lackland DT, Lewis TT, Lichtman JH, Longenecker CT, Loop MS, Lutsey PL, Martin SS, Matsushita K, Moran AE, Mussolino ME, Perak AM, Rosamond WD, Roth GA, Sampson UK, Satou GM, Schroeder EB, Shah SH, Shay CM, Spartano NL, Stokes A, Tirschwell DL, VanWagner LB, Tsao CW, American Heart Association Council on Epidemiology and Prevention Statistics Committee and Stroke Statistics Subcommittee (2020). Heart disease and stroke statistics—2020 update: a report from the American Heart Association. Circulation.

[6] Zeiler SR (2019). Should we care about early post-stroke rehabilitation? Not yet, but soon. Curr Neurol Neurosci Rep.

[7] Ostrowska PM, Śliwiński M, Studnicki R, Hansdorfer-Korzon R (2021). Telerehabilitation of post-stroke patients as a therapeutic solution in the era of the Covid-19 pandemic. Healthcare (Basel).

[8] Kleim JA, Jones TA (2008). Principles of experience-dependent neural plasticity: implications for rehabilitation after brain damage. J Speech Lang Hear Res.

[9] Maier M, Ballester BR, Verschure PFMJ (2019). Principles of neurorehabilitation after stroke based on motor learning and brain plasticity mechanisms. Front Syst Neurosci.

[10] Platz T (2021). Clinical Pathways in Stroke Rehabilitation: Evidence-Based Clinical Practice Recommendations.

[11] Cramer SC, Dodakian L, Le V, See J, Augsburger R, McKenzie A, Zhou RJ, Chiu NL, Heckhausen J, Cassidy JM, Scacchi W, Smith MT, Barrett AM, Knutson J, Edwards D, Putrino D, Agrawal K, Ngo K, Roth EJ, Tirschwell DL, Woodbury ML, Zafonte R, Zhao W, Spilker J, Wolf SL, Broderick JP, Janis S, National Institutes of Health StrokeNet Telerehab Investigators (2019). Efficacy of home-based telerehabilitation vs In-Clinic therapy for adults after stroke: a randomized clinical trial. JAMA Neurol.

[12] Rajsic S, Gothe H, Borba HH, Sroczynski G, Vujicic J, Toell T, Siebert U (2019). Economic burden of stroke: a systematic review on post-stroke care. Eur J Health Econ.

[13] Forman CR, Nielsen JB, Lorentzen J (2021). Neuroplasticity at home: improving home-based motor learning through technological solutions. a review. Front Rehabil Sci.

[14] Neibling BA, Jackson SM, Hayward KS, Barker RN (2021). Perseverance with technology-facilitated home-based upper limb practice after stroke: a systematic mixed studies review. J Neuroeng Rehabil.

[15] Maier M, Rubio Ballester B, Duff A, Duarte Oller E, Verschure PFMJ (2019). Effect of specific over nonspecific VR-based rehabilitation on poststroke motor recovery: a systematic meta-analysis. Neurorehabil Neural Repair.

[16] Ferreira B, Menezes P (2020). Gamifying motor rehabilitation therapies: challenges and opportunities of immersive technologies. Information.

[17] Selamat SNS, Che Me R, Ahmad Ainuddin H, Salim MSF, Ramli HR, Romli MH (2021). The application of technological intervention for stroke rehabilitation in southeast Asia: a scoping review with stakeholders' consultation. Front Public Health.

[18] Laver KE, Lange B, George S, Deutsch JE, Saposnik G, Crotty M (2017). Virtual reality for stroke rehabilitation. Cochrane Database Syst Rev.

[19] Gorman C, Gustafsson L (2022). The use of augmented reality for rehabilitation after stroke: a narrative review. Disabil Rehabil Assist Technol.

[20] Sousa ASP, Moreira J, Silva C, Mesquita I, Macedo R, Silva A, Santos R (2022). Usability of functional electrical stimulation in upper limb rehabilitation in post-stroke patients: a narrative review. Sensors (Basel).

[21] Peretti A, Amenta F, Tayebati SK, Nittari G, Mahdi SS (2017). Telerehabilitation: review of the state-of-the-art and areas of application. JMIR Rehabil Assist Technol.

[22] Chen J, Sun D, Zhang S, Shi Y, Qiao F, Zhou Y, Liu J, Ren C (2020). Effects of home-based telerehabilitation in patients with stroke: a randomized controlled trial. Neurology.

[23] Nikolaev VA, Nikolaev AA (2022). Recent trends in telerehabilitation of stroke patients: a narrative review. NeuroRehabilitation.

[24] Morse H, Biggart L, Pomeroy V, Rossit S (2022). Exploring perspectives from stroke survivors, carers and clinicians on virtual reality as a precursor to using telerehabilitation for spatial neglect post-stroke. Neuropsychol Rehabil.

[25] Zhou X, Du M, Zhou L (2018). Use of mobile applications in post-stroke rehabilitation: a systematic review. Top Stroke Rehabil.

[26] Chung BPH, Chiang WKH, Lau H, Lau TFO, Lai CWK, Sit CSY, Chan KY, Yeung CY, Lo TM, Hui E, Lee JSW (2020). Pilot study on comparisons between the effectiveness of mobile video-guided and paper-based home exercise programs on improving exercise adherence, self-efficacy for exercise and functional outcomes of patients with stroke with 3-month follow-up: a single-blind randomized controlled trial. Hong Kong Physiother J.

[27] Grau-Pellicer M, Lalanza J, Jovell-Fernández E, Capdevila L (2020). Impact of mHealth technology on adherence to healthy PA after stroke: a randomized study. Top Stroke Rehabil.

[28] Moher D, Liberati A, Tetzlaff J, Altman DG, PRISMA Group (2009). Preferred reporting items for systematic reviews and meta-analyses: the PRISMA statement. Ann Intern Med.

[29] Shea BJ, Reeves BC, Wells G, Thuku M, Hamel C, Moran J, Moher D, Tugwell P, Welch V, Kristjansson E, Henry DA (2017). AMSTAR 2: a critical appraisal tool for systematic reviews that include randomised or non-randomised studies of healthcare interventions, or both. BMJ.

[30] Rintala A, Päivärinne V, Hakala S, Paltamaa J, Heinonen A, Karvanen J, Sjögren T (2019). Effectiveness of technology-based distance physical rehabilitation interventions for improving physical functioning in stroke: a systematic review and meta-analysis of randomized controlled trials. Arch Phys Med Rehabil.

[31] Parker J, Powell L, Mawson S (2020). Effectiveness of upper limb wearable technology for improving activity and participation in adult stroke survivors: systematic review. J Med Internet Res.

[32] Phan HL, Le TH, Lim JM, Hwang CH, Koo K (2022). Effectiveness of augmented reality in stroke rehabilitation: a meta-analysis. Appl Sci.

[33] Gelineau A, Perrochon A, Robin L, Daviet J, Mandigout S (2022). Measured and perceived effects of upper limb home-based exergaming interventions on activity after stroke: a systematic review and meta-analysis. Int J Environ Res Public Health.

[34] Rintala A, Kossi O, Bonnechère B, Evers L, Printemps E, Feys P (2023). Mobile health applications for improving physical function, physical activity, and quality of life in stroke survivors: a systematic review. Disabil Rehabil.

[35] Szeto SG, Wan H, Alavinia M, Dukelow S, MacNeill H (2023). Effect of mobile application types on stroke rehabilitation: a systematic review. J Neuroeng Rehabil.

[36] Hao J, Pu Y, Chen Z, Siu K (2023). Effects of virtual reality-based telerehabilitation for stroke patients: a systematic review and meta-analysis of randomized controlled trials. J Stroke Cerebrovasc Dis.

[37] Raghavendra P, Bornman J, Granlund M, Björck-Akesson Eva (2007). The World Health Organization's International Classification of Functioning, Disability and Health: implications for clinical and research practice in the field of augmentative and alternative communication. Augment Altern Commun.

[38] Daly JJ, McCabe JP, Holcomb J, Monkiewicz M, Gansen J, Pundik S (2019). Long-dose intensive therapy is necessary for strong, clinically significant, upper limb functional gains and retained gains in severe/moderate chronic stroke. Neurorehabil Neural Repair.

[39] Guggisberg AG, Koch PJ, Hummel FC, Buetefisch CM (2019). Brain networks and their relevance for stroke rehabilitation. Clin Neurophysiol.

[40] Coscia M, Wessel M, Chaudary U, Millán JDR, Micera S, Guggisberg A, Vuadens P, Donoghue J, Birbaumer N, Hummel FC (2019). Neurotechnology-aided interventions for upper limb motor rehabilitation in severe chronic stroke. Brain.

[41] Assis GAD, Corrêa AGD, Martins MBR, Pedrozo WG, Lopes RDD (2016). An augmented reality system for upper-limb post-stroke motor rehabilitation: a feasibility study. Disabil Rehabil Assist Technol.

[42] Bank PJM, Cidota MA, Ouwehand PEW, Lukosch SG (2018). Patient-tailored augmented reality games for assessing upper extremity motor impairments in Parkinson's disease and stroke. J Med Syst.

[43] Ballester BR, Nirme J, Camacho I, Duarte E, Rodríguez S, Cuxart A, Duff A, Verschure PFMJ (2017). Domiciliary VR-based therapy for functional recovery and cortical reorganization: randomized controlled trial in participants at the chronic stage post stroke. JMIR Serious Games.

[44] Nijenhuis SM, Prange-Lasonder GB, Stienen AH, Rietman JS, Buurke JH (2017). Effects of training with a passive hand orthosis and games at home in chronic stroke: a pilot randomised controlled trial. Clin Rehabil.

[45] Chen Y, Abel KT, Janecek JT, Chen Y, Zheng K, Cramer SC (2019). Home-based technologies for stroke rehabilitation: a systematic review. Int J Med Inform.

[46] Toh SFM, Chia PF, Fong KNK (2022). Effectiveness of home-based upper limb rehabilitation in stroke survivors: a systematic review and meta-analysis. Front Neurol.

[47] Lloréns R, Noé E, Colomer C, Alcañiz M (2015). Effectiveness, usability, and cost-benefit of a virtual reality-based telerehabilitation program for balance recovery after stroke: a randomized controlled trial. Arch Phys Med Rehabil.

[48] Keser Z, Ikramuddin S, Shekhar S, Feng W (2023). Neuromodulation for post-stroke motor recovery: a narrative review of invasive and non-invasive tools. Curr Neurol Neurosci Rep.

[49] Adeyemo BO, Simis M, Macea DD, Fregni F (2012). Systematic review of parameters of stimulation, clinical trial design characteristics, and motor outcomes in non-invasive brain stimulation in stroke. Front Psychiatry.

