# Supplementary material for: Poststroke eHealth Technologies–Based Rehabilitation for Upper Limb Recovery: Systematic Review
Source: J Med Internet Res. 2025 Mar 4;27:e57957. doi: 10.2196/57957 (PMC11920654; doi:10.2196/57957)
Supplement: Multimedia Appendix 1 [file jmir_v27i1e57957_app1.docx]

**Multimedia Appendix 1.** Strings used for the search in PubMed, Web of Science, Scopus and Embase databases, and Google Scholar

| post stroke outpatient OR after stroke outpatient AND at home upper limb telerehabilitation OR at home paretic arm telerehabilitation OR home eHealth rehabilitation OR smartphone-based rehabilitation  post stroke person OR after stroke person AND at home upper limb telerehabilitation OR at home paretic arm telerehabilitation OR home eHealth rehabilitation OR smartphone-based rehabilitation  post stroke people OR after stroke people AND at home upper limb telerehabilitation OR at home paretic arm telerehabilitation OR home eHealth rehabilitation OR smartphone-based rehabilitation  after hospital discharge patient OR after hospital discharge people AND at home upper limb telerehabilitation OR paretic arm telerehabilitation at home OR home eHealth rehabilitation OR smartphone-based rehabilitation |
| --- |
