# Supplementary material for: Poststroke eHealth Technologies–Based Rehabilitation for Upper Limb Recovery: Systematic Review
Source: J Med Internet Res. 2025 Mar 4;27:e57957. doi: 10.2196/57957 (PMC11920654; doi:10.2196/57957)
Supplement: Multimedia Appendix 2 [file jmir_v27i1e57957_app2.docx]

**Multimedia Appendix 2.** AMSTAR-2

| AMSTAR-2 items | | | |
| --- | --- | --- | --- |
| Item | Item type | Description | Responses |
| 1 | Non-critical | Did the research questions and inclusion criteria for the review include the components of PICO? | Yes |
|  |  |  | No |
| 2 | Critical | Did the report of the review contain an explicit statement that the review methods | Yes |
|  |  | were established prior to the conduct of the review and did the report justify any signiﬁcant deviations from the protocol? | Partial yes |
|  |  |  | No |
| 3 | Non-critical | Did the review authors explain their selection of the study designs for inclusion in the review? | Yes |
|  |  |  | No |
| 4 | Critical | Did the review authors use a comprehensive literature search strategy? | Yes |
|  |  |  | Partial yes |
|  |  |  | No |
| 5 | Non-critical | Did the review authors perform study selection in duplicate? | Yes |
|  |  |  | No |
| 6 | Non-critical | Did the review authors perform data extraction in duplicate? | Yes |
|  |  |  | No |
| 7 | Critical | Did the review authors provide a list of excluded studies and justify the exclusions? | Yes |
|  |  |  | Partial yes |
|  |  |  | No |
| 8 | Non-critical | Did the review authors describe the included studies in adequate detail? | Yes |
|  |  |  | Partial yes |
|  |  |  | No |
| 9 | Critical | Did the review authors use a satisfactory technique for assessing the risk of bias (RoB) in individual studies that were included in the review? | Yes |
|  |  |  | Partial yes |
|  |  |  | No |
|  |  |  | Includes only NRSI/RCTs |
| 10 | Non-critical | Did the review authors report on the sources of funding for the studies included in the review? | Yes |
|  |  |  | No |
| 11 | Critical | If meta-analysis was performed, did the review authors use appropriate methods for statistical combination of results? | Yes |
|  |  |  | No |
|  |  |  | No M-A conducted |
| 12 | Non-critical | If meta-analysis was performed, did the review authors assess the potential impact of RoB in individual studies on the results of the meta-analysis or other evidence synthesis? | Yes |
|  |  |  | No |
|  |  |  | No M-A conducted |
| 13 | Critical | Did the review authors account for RoB in individual studies when interpreting/ discussing the results of the review? | Yes |
|  |  |  | No |
| 14 | Non-critical | Did the review authors provide a satisfactory explanation for, and discussion of, any heterogeneity observed in the results of the review? | Yes |
|  |  |  | No |
| 15 | Critical | If they performed quantitative synthesis, did the review authors carry out an adequate investigation of publication bias (small study bias) and discuss its likely impact on the results of the review? | Yes |
|  |  |  | No |
|  |  |  | No M-A conducted |
| 16 | Non-critical | Did the review authors report any potential sources of conﬂict of interest, including any funding they received for conducting the review? | Yes |
|  |  |  | No |
| AMSTAR-2 criteria for overall conﬁdence | | | |
| High conﬁdence | | ≤1 non-critical weakness | - |
| Moderate conﬁdence | | >1 non-critical weakness | - |
| Low conﬁdence | | 1 critical flaw, with or without non-critical weaknesses | - |
| Critically low conﬁdence | | >1 critical flaw, with or without non-critical weaknesses | - |

|  | Q1 | Q2 | Q3 | Q4 | Q5 | Q6 | Q7 | Q8 | Q9 | Q10 | Q11 | Q12 | Q13 | Q14 | Q15 | Q16 | Confidence Level |
| --- | --- | --- | --- | --- | --- | --- | --- | --- | --- | --- | --- | --- | --- | --- | --- | --- | --- |
| Aki Rintala et al., 2019 [31] | yes | yes | yes | partial yes | yes | yes | no | yes | yes RCTs / includes only RCTs | no | yes RCTs | yes | yes | yes | yes | no | LOW CONFIDENCE |
| Jack Parker et al., 2020 [32] | yes | partial yes | yes | partial yes | yes | yes | no | partial yes | yes RCTs / yes NRSI | no | no meta-analysis  conducted | no meta-analysis  conducted | yes | no | no meta-analysis  conducted | yes | CRITICALLY LOW CONFIDENCE |
| Huu Lam Phan et al., 2022 [33] | yes | partial yes | yes | partial yes | yes | no | no | partial yes | yes RCTs / yes NRSI | no | yes RCTs / no NRSI | no | no | yes | yes | yes | CRITICALLY LOW CONFIDENCE |
| Axelle Gelineu et al., 2022 [34] | yes | yes | yes | partial yes | yes | yes | no | yes | yes RCTs / includes only RCTs | yes | yes RCTs | yes | yes | yes | no | yes | CRITICALLY LOW CONFIDENCE |
| A. Rintala et al., 2022 [35] | yes | partial yes | yes | partial yes | yes | no | no | yes | yes RCTs / yes NRSI | no | no meta-analysis  conducted | no meta-analysis  conducted | yes | yes | no meta-analysis  conducted | yes | CRITICALLY LOW CONFIDENCE |
| Stephen G. Szeto et al., 2023 [36] | no | partial yes | yes | partial yes | yes | no | no | partial yes | yes RCTs / no NRSI | no | no meta-analysis  conducted | no meta-analysis  conducted | yes | no | no meta-analysis  conducted | yes | CRITICALLY LOW CONFIDENCE |
| Jie Hao et al., 2023 [37] | no | no | yes | partial yes | yes | no | no | yes | no RCTs / includes only RCTs | no | yes RCTs | no | no | yes | no | yes | CRITICALLY LOW CONFIDENCE |
