# Supplementary material for: Poststroke eHealth Technologies–Based Rehabilitation for Upper Limb Recovery: Systematic Review
Source: J Med Internet Res. 2025 Mar 4;27:e57957. doi: 10.2196/57957 (PMC11920654; doi:10.2196/57957)
Supplement: Multimedia Appendix 3 [file jmir_v27i1e57957_app3.docx]

**Multimedia Appendix 3.** Characteristics of the reviewed studies

| Reference | Study objective | Inclusion criteria | Search Strategy | | Intervention  tested | | Methodological  quality | Findings |
| --- | --- | --- | --- | --- | --- | --- | --- | --- |
| Authors/  Years |  |  | Search database and date | Number of articles reviewed | Technology | Type of Studies  Participants | Study quality assessment scale used |  |
| Aki Rintala et al., 2019 [31] | It investigated the effectiveness of technology-based  distance physical rehabilitation interventions on physical  functioning compared to a combination of traditional treatments  such as similar treatment, other treatment, and usual care in  persons with stroke. | PICOS structure: (P) persons with stroke; (I) any  technology  used to monitor, promote, or increase  physical functioning as a distance physical rehabilitation intervention;  (C) any control group not receiving rehabilitation intervention  or receiving rehabilitation intervention  without the use of technology; (O)  outcome measures of physical functioning; and (S) RCTs that  were published in English, Finnish, Swedish, or German. | Cochrane Central Register of Controlled Trials, Cumulative  Index to Nursing and Allied Health Literature, Excerpta  Medica Database, Database of the National Library of Medicine,  Physiotherapy Evidence Database, and Web of Science.  Studies published between January 2000-May 2018. | 13 | Online video monitoring; telephone calls; exercise videos (tablet/DVD); virtual training program; physical exercise programs + gamification. | RCTs.  605 patients with a mean age of  65.2 years. | Physiotherapy Evidence Database (PEDro) Scale. | Technology-based distance physical rehabilitation  interventions had a similar effect on physical functioning outcomes  of ADL, upper and lower extremity functioning, balance,  physical activity, and participation, when compared to the combinations  of traditional treatments not involving the use of technology. |
| Jack Parker et al., 2020 [32] | Assess the effectiveness of Upper Limb wearable technology for improving activity and participation  in adult stroke survivors. | PICOS structure: (P)  poststroke adults, (I) technological interventions  for Upper Limb rehabilitation in stroke survivors, (C) included studies  included a comparison group and were not limited to randomized  controlled trials (RCTs),  (O) activity and  participation measures of Upper Limb function poststroke,  (S) Studies reporting randomized controlled trials or randomized comparable trials and studies measuring activity and or participation as classified by the World Health  Organization’s International Classification of Functioning, Disability and Health (ICF-WHO)  Framework. | MEDLINE, Web of Science (Core collection),  CINAHL, Scopus, and the Cochrane Library. Medical Subject  Headings (MeSH).  Studies published between 2000 and April 2019. | 11 | Wearable and portable technology that measures or monitors activity. | RCTs, non RCTs.  354 patients with a mean age of  57 years. | Cochrane Risk of Bias (CRoB) for RCTs and Downs and Black Instrument for non-RCTs. | There is little evidence in the literature  to support the use of wearable technologies to improve activity  and participation for independent UL rehabilitation following  a stroke. |
| Huu Lam Phan et al., 2022 [33] | Evaluate the effectiveness of the AR for the upper and lower limb functional recovery after stroke. | PICO structure: (P) stroke patients, (I) AR, (C) comparison with baseline or conventional therapy, (O) activity and  participation measures of Upper Limb and Lower Limb function poststroke as classified by the World Health  Organization’s International Classification of Functioning, Disability and Health (ICF-WHO)  Framework. | PubMed (Medline), Web of Science (WOS), Science Direct, Embase, and SAGE  Publication.  Studies published between 2010-2021. | 13 | AR-based rehabilitation | RCTs  and observational studies.  199 patients  with a mean age range of 53.7 years. | QualSyst. | AR applications  could offer options for increasing treatment intensity and promoting motor recovery after a  stroke. |
| Axelle Gelineu et al., 2022 [34] | Examine the measured  and perceived effects of UL home-based exergaming interventions on activity after stroke,  compared with conventional therapy, in post-intervention and follow-up. | PICOS structure:  (P) Post-stroke adults, (I) Intervention using an exergaming  technology for UL rehabilitation at home, (C) Comparison with conventional therapy (i.e.,  usual practice), (O) Outcome measures used to assess activities by observation and self-reporting,  (S) Study design included only Randomized Controlled Trials. | Cochrane Central Register of Controlled  Trials (CENTRAL; Cochrane Library), MEDLINE (PubMed search engine), Cumulative  Index to Nursing and Allied Health Literature (CINAHL), EMBASE, and SCOPUS online  Databases.  Studies published before July 2021. | 9 | Five studies used nonspecific video  game systems (e.g., the Nintendo Wii™, the Microsoft Xbox Kinect™, the  Sony PlayStation EyeToy™). Some of the video-game systems have been combined  with a system specifically designed for rehabilitation  with a pair of data gloves, Wiimotes™ with a virtual glove, and Music-  Glove. Others used a specific rehabilitation device; 3D motion tracking system; SCRIPT  dynamic wrist and hand orthosis and SaeboMAS. | RCTs.    535 stroke patients. | Physiotherapy Evidence Database (PEDro) Scale. | Upper limb home-based exergaming interventions  were no more effective in terms of activity than conventional therapy after stroke, according to  the observational and subjective assessments in post-intervention and follow-up. |
| A. Rintala et al., 2022 [35] | Evaluate the effectiveness of mobile health applications (mHealth apps) containing a physical  training component on physical function and physical activity in stroke rehabilitation. | PICOS structure:  (P) adult poststroke  survivors in any stages who experienced an ischemic or  hemorrhagic stroke, (I) intervention using a  mHealth app on a smartphone or a tablet containing any type of  physical training component  for the person to use the app independently  of a therapist but the help of a caregiver was allowed,  (C) any type of control group (if applicable), (O) any type of outcome  measures of PF or PA. A secondary outcome related to QoL  was extracted if QoL was a subject of investigation in studies  including PF or PA, (S) randomized controlled trials (RCTs), nonrandomized  clinical trials (non-RCTs), or uncontrolled clinical trials  published in English. | Pubmed, Web of Science, and Scopus.  Studies published  from inception to  12 July 2022. | 11 | mHealth app on a smartphone or a tablet containing any type of  physical training component (e.g., physical exercises, physical  therapy, or physical activity) | RCTs, non-RCTs, or uncontrolled clinical trials.  264 stroke survivors with a mean age of 59.3 years. | Physiotherapy Evidence Database (PEDro) Scale for RCTs and Downs and Black checklist for non RCTs. | Six out of seven studies  reported statistically significant improvements in physical function in favor of the experimental group,  with the most robust findings for upper extremity function. For physical activity, statistically significant  improvements were seen in the experimental groups. |
| Stephen G. Szeto et al., 2023 [36] | Review the effect of mobile  apps for stroke rehabilitation on stroke-related impairments  and functional outcomes. | (1) Randomized control trials (RCTs),  quasi-experimental clinical trials, or qualitative studies,  (2) study population were adult (18 + years of age) stroke  survivors who  underwent rehabilitation, and (3) the primary  intervention studied was a mobile app. | MEDLINE, EMBASE,  Cochrane Library, CINAHL, SCOPUS, COMPENDEX,  and IEEE Xplore.  Studies published prior to May 31, 2020. | 29 | Mobile Apps (phone, tablet,  or PC) for stroke rehabilitation on any operating system (e.g., iOS, Android,  Windows) | RCTs and quasi-experimental studies.  778 patients with a mean age of 58.7 years. | Cochrane  Risk of Bias tool (CRoB), Downs and Black checklist. | Mobile  apps can be used to improve stroke rehabilitation, particularly  in combination with face-to-face therapy for  motor paresis, aphasia, and adherence to exercise. |
| Jie Hao et al., 2023 [37] | Gather and summarize evidence  of the use of virtual reality-based telerehabilitation for  patients after stroke and compare it with conventional in-person  rehabilitation. | (1) participants were adult patients  (age >= 18 years old) with the diagnosis of stroke; (2) the  combination of virtual reality and telerehabilitation was  used for intervention, both commercial and customized  virtual reality systems were included; (3) the study type  was a randomized controlled trial; (4) the articles were  peer-reviewed. | PubMed, Embase, PsycINFO, IEEE Explore,  Cumulative Index of Nursing and Allied Health, and Scopus.  Studies published between 2006-2021. | 9 | Synchronous and asynchronous virtual reality-based telerehabilitation system. | RCTs.  260 participants. | Physiotherapy Evidence Database (PEDro) Scale. | Compared with conventional in-person rehabilitation,  the meta-analysis indicated that virtual reality-based telerehabilitation had  comparable outcomes of upper extremity function and balance function. Both  groups demonstrated similar effects on outcomes in mobility, cognition, activities  of daily life, and quality of life. |
