# Supplementary material for: Poststroke eHealth Technologies–Based Rehabilitation for Upper Limb Recovery: Systematic Review
Source: J Med Internet Res. 2025 Mar 4;27:e57957. doi: 10.2196/57957 (PMC11920654; doi:10.2196/57957)
Supplement: Multimedia Appendix 4 [file jmir_v27i1e57957_app4.docx]

**Multimedia Appendix 4.** Outcome measures

| Outcome | Assessment type | Outcome measures | Assessment area/target |
| --- | --- | --- | --- |
| Upper Limb (UL) function |  |  |  |
|  | *PATIENT REPORTED OUTCOME* | Late-Life Function and Disability Instrument (LLFDI) [1] | Functional limitations/disability (frequency and limitation in performing life tasks)   - Difficulty perceived - UE Function |
|  |  | Motor Activity Log (MAL) [2] | UE function and rehabilitation outcome after stroke (quality and amount of movement during ADLs)   - Stroke specific scale - Difficulty perceived - UE Function - Fine manual dexterity - Gross manual dexterity |
|  |  | Disabilities of the arm, shoulder and hand (DASH) questionnaire [3] | Musculoskeletal impairments of UL (symptomps and ability of UE from patients' perspective)   - Pain - Strenght - Difficulty perceived - Motor impairment - Fine manual dexterity - Gross manual dexterity |
|  |  | Quick Disabilities of Arm, Shoulder & Hand – DASH short version (QuickDASH) [4] | Musculoskeletal impairments of UL (physical function and symptoms from patients' POV)   - Pain - Difficulty perceived - Motor impairment - Fine manual dexterity - Gross manual dexterity |
|  |  | Canadian Occupational Performance Measure (COPM) [5] | Motor/occupational performance (ADLs, functional mobility, life participation)   - Difficulty perceived - Motor impairment |
|  |  | ABILHAND scale [6] | UE function, manual ability (dexterity on ADLs from patients' POV)   - Stroke specific scale - Difficulty perceived - UE Function - Fine manual dexterity - Gross manual dexterity |
|  | *PERFORMANCE MEASURE* | Fugl-Meyer Assessment – Upper Extremity (FMA-UE) scale [7] | Sensorimotor impairment (motor function, ROM, coordination, reflex, joint pain, sensation)   - Stroke specific scale - Pain - Strenght - Motor impairment - Fine manual dexterity - Gross manual dexterity |
|  |  | Wolf Motor Function Test (WMFT) [8] | UE performance (dexterity and fluidity in timed functional movements)   - Stroke specific scale - UE Function - Fine manual dexterity - Gross manual dexterity |
|  |  | Manual Function Test (MFT) [9] | Dexterity after stroke (arm motion and manipulation)   - Stroke specific scale - UE Function - Fine manual dexterity - Gross manual dexterity |
|  |  | Box and Block Test (BBT) [10] | Motor ability after stroke (dexterity, coordination)   - Stroke specific scale - UE Function - Gross manual dexterity |
|  |  | Action Research Arm Test (ARAT) [11] | UE function (coordination, dexterity)   - Stroke specific scale - UE Function - Fine manual dexterity - Gross manual dexterity |
|  |  | Chedoke Arm and Hand Activity Inventory (CAHAI) [12] | UE impairment and functional recovery after stroke (real-life functional tasks)   - Stroke specific scale - Motor impairment - Fine manual dexterity - Gross manual dexterity |
|  |  | Jebsen-Taylor Hand Function Test (JTHFT) [13] | UE function (timed hand functions for ADLs)   - UE Function - Fine manual dexterity - Gross manual dexterity |
|  |  | Upper Extremity Function Test (UEFT) [14] | UE functional impairment and severity   - Motor impairment - Fine manual dexterity - Gross manual dexterity |
|  |  | Ashworth scale (AS) and Modified Ashworth Scale (MAS) [15] | Increase in muscle tone   - Stroke specific scale - Spasticity |
|  |  | Stroke Upper Limb Capacity Scale (SULCS) [16] | UE function after stroke (daily tasks in the acute rehabilitation phase)   - Stroke specific scale - UE Function - Fine manual dexterity - Gross manual dexterity |
|  |  | Nine Hole Peg Test (NHPT) [17] | UE function (finger dexterity)   - Fine manual dexterity |
|  |  | Barthel Index (BI) [18] | Index of independence/disability level (ADLs, functional mobility, gait, NO specific for UE)   - Motor impairment |
|  |  | Grooved Pegboard Test (GPT) [19] | Manipulation, dexterity   - Fine manual dexterity |
|  |  | Purdue Pegboard Test (PPT) [20] | Manipulative dexterity, coordination   - Fine manual dexterity - Gross manual dexterity |
|  |  | Manual Muscle Test – Upper Extremity (MMT-UE) [21] | Muscle strength and function (muscle group of shoulder, elbow, forearm, wrist)   - Strenght - UE Function |
|  |  | Brunnstrom stage – Upper Extremity (B-stage-UE) [22] | Motor function and recovery process stages   - Stroke specific scale - Spasticity - Motor impairment |
|  |  | Range of motion (ROM)* [23] | Total joint movement (impairment and rehabilitation progress)   - UE Function |
|  |  | Wrist Extension (WE) and  Wrist Flexion (WF) [24]  Finger Extension (FE) and Finger Flexion (FF) [25] | *Specific ROM (range of motion)   - UE Function |
|  |  | Motricity Index (MI) [26] | Limb strenght and functional mobility   - Strenght - UE Function |

## **References**

1. Jette AM, Haley SM, Coster WJ, Kooyoomjian JT, Levenson S, Heeren T et al. Late life function and disability instrument: I. development and evaluation of the disability component. J Gerontol A Biol Sci Med Sci 2002;57(4):M209-M216. [FREE Full text] [doi: 10.1093/gerona/57.4.M209] [Medline: 11909885]
2. Uswatte G, Taub E, Morris D, Vignolo M, McCulloch K. Reliability and validity of the upper-extremity motor activity log-14 for measuring real-world arm use. Stroke 2005;36(11):2493-2496. [FREE Full text] [doi: 10.1161/01.STR.0000185928.90848.2e] [Medline: 16224078]
3. Jester A, Harth A, Wind G, Germann G, Sauerbier M. Disabilities of the arm, shoulder and hand (DASH) questionnaire: determining functional activity profiles in patients with upper extremity disorders. J Hand Surg Br 2005;30(1):23-28. [FREE Full text] [doi: 10.1016/J.JHSB.2004.08.008] [Medline: 15620487]
4. Gummesson C, Ward MM, Atroshi I. The shortened disabilities of the arm, shoulder and hand questionnaire (QuickDASH): validity and reliability based on responses within the full-length DASH. BMC Musculoskelet Disord 2006;7(1):1-7. [FREE Full text][doi: 10.1186/1471-2474-7-44] [Medline: 16709254]
5. Carswell A, McColl MA, Baptiste S, Law M, Polatajko H, Pollock N. The Canadian occupational performance measure: a research and clinical literature review. Can J Occup Ther 2004 Oct;71(4):210-222. [FREE Full text] [doi: 10.1177/000841740407100406] [Medline: 15586853]
6. Penta M, Thonnard J-L, Tesio L. ABILHAND: a Rasch-built measure of manual ability. Arch Phys Med Rehabil 1998;79(9):1038-1042. [FREE Full text] [doi: 10.1016/S0003-9993(98)90167-8] [Medline: 9749680]
7. Fugl-Meyer AR, Jaasko L, Leyman I, Olsson S, Steglind S. The post-stroke hemiplegic patient1, a method for evaluation of physical performance. Scand J Rehabil Med 1975;7(1):13-31. [FREE Full text] [doi: 10.2340/1650197771331] [Medline: 1135616]
8. Wolf SL, Catlin PA, Ellis M, Archer AL, Morgan B, Piacentino A. Assessing Wolf motor function test as outcome measure for research in patients after stroke. Stroke 2001 Jul;32(7):1635-1639. [FREE Full text] [doi: 10.1161/01.STR.32.7.1635] [Medline: 11441212]
9. Michimata A, Kondo T, Suzukamo Y, Chiba M, Izumi SI. The manual function test: norms for 20-to 90-year-olds and effects of age, gender, and hand dominance on dexterity. Tohoku J Exp Med 2008;214(3):257-267. [FREE Full text] [doi: 10.1620/tjem.214.257] [Medline: 18323695]
10. Mathiowetz V, Volland G, Kashman N, Weber K. Adult norms for the box and block test of manual dexterity. Am J Occup Ther 1985;39(6):386-391. [FREE Full text] [doi: 10.5014/ajot.39.6.386] [Medline: 3160243]
11. Yozbatiran N, Der-Yeghiaian L, Cramer SC. A standardized approach to performing the action research arm test. Neurorehabil Neural Repair 2008;22(1):78-90. [FREE Full text] [doi: 10.1177/1545968307305353] [Medline: 17704352]
12. Barreca SR, Stratford PW, Lambert CL, Masters LM, Streiner DL. Test-retest reliability, validity, and sensitivity of the Chedoke arm and hand activity inventory: a new measure of upper-limb function for survivors of stroke. Arch Phys Med Rehabil 2005;86(8):1616-1622. [FREE Full text] [doi: 10.1016/j.apmr.2005.03.017] [Medline: 16084816]
13. Jebsen RH, Taylor N, Trieschmann R, Trotter M, Howard L. An objective and standardized test of hand function. Arch phys med Rehabil 1969;50(6):311-319. [PMID:5788487]
14. Carroll D. A quantitative test of upper extremity function. J Chronic Dis 1965;18(5):479-491. [FREE Full text] [doi: 10.1016/0021-9681(65)90030-5] [Medline: 14293031]
15. Dunning, K. Ashworth spasticity scale (and modified version). In: Kreutzer JS, DeLuca J, Caplan B, editors. Encyclopedia of clinical neuropsychology. New York: Springer; 2011. p. 254-255.
16. Roorda LD, Houwink A, Smits W, Molenaar IW, Geurts AC. Measuring upper limb capacity in poststroke patients: development, fit of the monotone homogeneity model, unidimensionality, fit of the double monotonicity model, differential item functioning, internal consistency, and feasibility of the stroke upper limb capacity scale, SULCS. Arch Phys Med Rehabil 2011;92(2):214-227. [FREE Full text] [doi: 10.1016/j.apmr.2010.10.034] [Medline: 21272717]
17. Kellor M, Frost J, Silberberg N, Iversen I, Cummings R. Hand strength and dexterity. Am J Occup Ther 1971 Mar;25(2):77-83. [PMID:5551515]
18. Mahoney FI, Barthel DW. Functional evaluation: the Barthel index: a simple index of independence useful in scoring improvement in the rehabilitation of the chronically ill. Md State Med J 1965 Feb;14:61-65. [PMID:14258950]
19. Merker B, Podell K. Grooved pegboard test. In: Kreutzer JS, DeLuca J, Caplan B, editors. Encyclopedia of clinical neuropsychology. New York: Springer; 2011.
20. Tiffin J, Asher EJ. The purdue pegboard: norms and studies of reliability and validity. J Appl Psychol 1948;32(3):234-247. [doi: 10.1037/h0061266]
21. Roman NA, Miclaus RS, Nicolau C, Sechel G. Customized manual muscle testing for post-stroke upper extremity assessment. Brain Sci 2022 Mar 28;12(4):457. [FREE Full text] [doi: 10.3390/brainsci12040457] [Medline: 35447988]
22. Huang CY, Lin GH, Huang YJ, Song CY, Lee YC, How MJ, Chen YM, Hsueh IP, Chen MH, Hsieh CL. Improving the utility of the Brunnstrom recovery stages in patients with stroke: validation and quantification. Medicine (Baltimore) 2016 Aug;95(31):e4508. [FREE Full text] [doi: 10.1097/MD.0000000000004508] [Medline: 27495103]
23. Gajdosik RL, Bohannon RW. Clinical measurement of range of motion: review of goniometry emphasizing reliability and validity. Phys Ther 1987;67(12):1867-1872. [FREE Full text] [doi: 10.1093/ptj/67.12.1867] [Medline: 3685114]
24. Norkin CC, White DJ. Measurement of Joint Motion: A Guide to Goniometry. 5th edition. Philadelphia, Pa: FA Davis Company; 2016. ISBN:9780803645660
25. Park J, Xu D. Multi-finger interaction and synergies in finger flexion and extension force production. Front Hum Neurosci 2017 Jun 19;11:318. [FREE Full text] [doi: 10.3389/fnhum.2017.00318] [Medline: 28674489]
26. Demeurisse G, Dermol O, Robaye E. Motor evaluation in vascular hemiplegia. Eur Neurol 1980;19(6):382-389. [doi: 10.1159/000115178] [Medline: 7439211]
